# Systematic Review of Methods for Individual Prediction of Postoperative Pain

**DOI:** 10.1155/prm/1331412

**Published:** 2025-01-25

**Authors:** Krister Mogianos, Jonas Åkeson, Anna K. M. Persson

**Affiliations:** ^1^Department of Anesthesiology and Intensive Care Medicine, Halland's Hospital Halmstad, Halmstad, Sweden; ^2^Department of Clinical Sciences Malmö, Lund University, Malmö, Sweden; ^3^Department of Anesthesiology and Intensive Care Medicine, Skåne University Hospital, Malmö, Sweden

**Keywords:** acute postoperative pain, pain sensitivity, persistent postoperative pain, postoperative pain, postsurgical pain, prediction of postoperative pain

## Abstract

**Background:** Acute postoperative pain is a common problem in clinical practice and merits attention considering its potential long-term adverse effects. This systematic review covers current knowledge on methods for individual prediction of postoperative pain.

**Methods:** A systematic literature search was conducted using the PubMed, EMBASE, and CINAHL databases for original studies with adult patients published in English between 2016 and 2022. Inclusion required assessment of risk factors preoperatively and assessment of postoperative pain. No reviews, meta-analyses, or study protocols were included, nor studies with outcomes other than pain or where risk factor analysis was not performed preoperatively. A two peer-reviewed system was utilized using the screening and data collection tool Covidence, with a focus on new tools for preoperative pain prediction. The results were only analyzed qualitatively.

**Results:** The search yielded 1950 abstracts to be screened. In total, 208 articles were subjected to full-text review, and 107 articles were included in the data synthesis of this review. The evaluated scientific methods were grouped and analyzed separately. Psychometric questionnaires and methods for quantitative sensory testing are still being studied. New methods proposed include the evaluation of pain induced by tourniquet inflation, venous cannulation, or pin-prick stimulation, the analgesia/nociception index, electroencephalographic recording, and other new equipment developed for this purpose.

**Conclusion:** Various screening methods have been proposed to identify patients prone to postoperative pain. The focus has shifted from procedure-specific to individualized strategies to improve early management of pain. However, many traditional predictive methods still have a questionable role in clinical practice.

**Trial Registration:** ClinicalTrials.gov identifier: CRD42022298479

## 1. Background

Known risk factors for acute postoperative pain (APOP) are female sex, smoking, young age, preoperative pain and opioid consumption, and psychosocial factors such as anxiety and depression, functional disability, certain types of surgery (specifically surgery involving nerve damage), and a large skin incision [1–8]. Although young age is a risk factor for postoperative pain, high frailty in older patients is associated with the risk of developing persistent postoperative pain (PPOP) [9]. None of these risk factors are strong enough to be used alone for individual prediction of the risk for severe APOP. Various preoperative screening methods have been proposed to identify patients prone to severe APOP, and focus has shifted from procedure-specific to individualized strategies to improve early management of APOP and/or PPOP [10, 11]. Historically, the methods proposed and most extensively studied are psychometric evaluations, quantitative sensory thresholds (QSTs), genetics, and methods combining individual risk factors with these evaluations [8, 12].

Approximately 80% of patients who undergo surgery experience acute pain, less than half of whom report adequate pain relief [13], and persistent pain, i.e., pain continuing for more than 3 months after surgery, affects 10%–50% of patients [6, 14]. Despite recent advances in pain prediction and treatment, APOP remains insufficiently relieved [15]. Improved management of this common surgical complication is crucial for preventing stress, promoting early recovery, and reducing the risk of potentially harmful complications [16]. No predictive model has yet shown good enough results in combination with feasibility to merit use in standard anesthesia routines.

This systematic review was designed to compile current knowledge on methods recently proposed for the individual prediction of acute and PPOP intensity.

## 2. Methods

This systematic review was carried out in accordance with the Preferred Reporting Items for Systematic Reviews (PRISMA) guidelines. The search strategy and inclusion criteria were registered in the PROSPERO database on December 15, 2021, and published on January 15, 2022. The search strategy was developed in accordance with the PICO format in the PubMed database and was carried out on December 15, 2021, based on relevant MeSH and free-text terms. The search terms were adapted and used for the PubMed, EMBASE (Elsevier), and CINAHL Complete (EbscoHost) databases. Manual searches of the reference lists of the included papers were also carried out.

Covidence, a systematic screening and data collection tool, was used to perform literature searches to screen abstracts and subsequently review full texts. We included original randomized controlled trials and retrospective and prospective observational studies published in English between 2016 and 2022, comprising adult patients who underwent surgery regardless of country and for whom preoperative factors in combination with the intensity of postoperative acute or persistent pain and/or the use of postoperative analgesics were reported. We considered all studies with an association between preoperative factors and postoperative pain. Unpublished studies, congress abstracts, review articles, meta-analyses, or letters to the editor were considered ineligible, as were studies using postoperative outcomes more vaguely associated with postoperative pain per se, i.e., postoperative recovery, eating habits, and synovitis. There are multiple definitions of both APOP and PPOP, and in this review, we decided to include all since we believe there is value in trying to sum up current knowledge despite differences in methods.

Two researchers (K.M. and A.P.) independently screened abstracts of papers obtained in the original search for potential eligibility. Any abstract deemed relevant by at least one researcher was included for full-text assessment. The same two researchers then independently assessed the full texts of the articles with respect to the inclusion and exclusion criteria. Due to the large number of full-text articles, assessments of study quality and bias were conducted on a case-by-case basis without a specific tool, by looking at confounding factors, information bias, and selection bias. This was performed independently by the reviewers, and when conflicts arose, a consensus was reached after discussion. Both reviewers had to consent to include the article in the final systematic review. No meta-analyses or other summary measures were carried out. For a better overview, and due to category heterogeneity, the methods reported were categorized into psychological methods, quantitative sensory testing, combined, genetic, biochemical, and “other techniques” for the prediction of pain. The category structure was decided on post hoc. No effect measures were used to compare studies; rather, the results were analyzed qualitatively. For the qualitative comparison of results, we present whichever outcome measure the specific study used as the primary outcome.

## 3. Results

### 3.1. Literature Search

**Figure 1:**
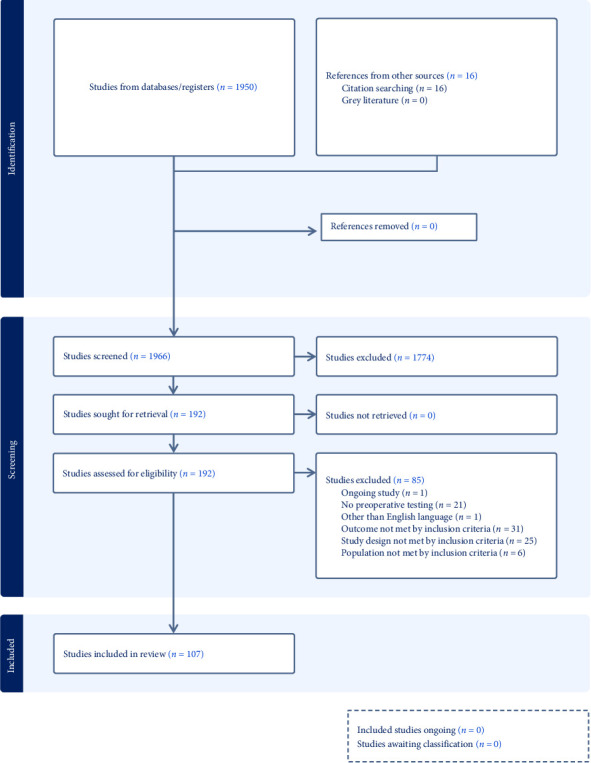
Consort diagram.

The search terms used identified 1950 items, of which 192 articles were selected for full-text review, and an additional 16 relevant studies were added after citation screening. One study protocol was excluded as well as studies without any actual preoperative test method (*n* = 21), not written in English (*n* = 1), outcomes not being postoperative pain or opioid consumption (*n* = 31), study design not met by the inclusion criteria (*n* = 25), and including pediatric population (*n* = 6). After exclusion, 107 studies passed full-text screening and were included in this systematic review (Figure 1).

### 3.2. Psychological Methods

#### 3.2.1. Psychometric Evaluation

The *Hospital Anxiety and Depression Scale* (HADS) has been used in research on pain prediction with conflicting results, and except for one study [17], its ability to predict PPOP has recently failed to be confirmed statistically. For APOP, the HADS score has been reported to be associated with pain intensity [18–22]. However, one study failed to show an association between acute pain and HADS after total knee arthroplasty [23].

Another psychometric assessment for depression only revealed an association between preoperative depression and severe acute pain after cardiac surgery [24]. In the same study, the preoperative depression score showed only weak correlations with APOP. Furthermore, the *Beck Depression Inventory II* Questionnaire failed to associate depression with PPOP in a mixed surgery cohort [25].

Regarding the *Pain Catastrophizing Scale* (PCS) [26], one of the most studied psychometrics in the current systematic review, results remain contradictory (Table 1). Several recent studies have correlated PCS with acute pain after cardiac surgery [27], upper extremity surgery [28], breast cancer surgery [29, 30], and high pain trajectories following major elective surgery [31]. Furthermore, studies also suggest that PCS is associated with persistent pain after total knee arthroplasty [32], hysterectomy [33], mixed surgery [34], and breast cancer surgery [35]. However, the PCS has not been found to predict pain in a large number of other recent studies [29, 36–44]. The PCS has shown better prediction of postoperative pain when related to experimental pain, compared to just any pain experience [45].

One study examined alexithymia, the inability to identify emotions within oneself and others, in patients undergoing bariatric surgery. In this study, patients who were positive for alexithymia had higher APOP and postoperative opioid consumption [46] (Table 1).

#### 3.2.2. Self-Evaluation

Self-expected extent of pain after surgery has been found to predict moderate-to-severe acute pain after thoracoscopic surgery [37]. Furthermore, the individual ability to forecast postoperative pain and disability has been found to correlate with actual levels [28], and with more acute pain after mixed surgery [20], and breast cancer surgery [47]. Self-evaluation has also been linked to levels of PPOP [40, 48] (Table 1).

#### 3.2.3. Evaluation of Pain Sensitivity

Scores obtained with the *Pain Sensitivity Questionnaire* (PSQ) have been reported to correlate with acute and persistent pain after cardiac surgery [49] and APOP within 24 h [50], whereas this correlation was not seen for persistent pain [33, 48].

The *Pain Self-Efficacy Questionnaire* (PSEQ) has been reported to independently predict acute pain after various kinds of surgery [51], whereas no correlation was found after joint arthroplasty [42].

The *Central Sensitization Inventory* (CSI) was developed to assess central sensitization [52]. In patients who underwent revision hip surgery, CSI was associated with persistent pain up until 2 years [53]. This result is consistent with those found in larger cohorts undergoing large joint arthroplasty and breast cancer surgery [35, 41, 42] (Table 1).

#### 3.2.4. Evaluation of Anxiety and Stress

Feeling anxious has been associated with severe APOP, as has feeling helpless because of pain [54]. Furthermore, preoperative emotional state predicts negative expectations and acts as a mediator of acute pain in patients undergoing knee arthroplasty [55].

The *State-Trait Anxiety Inventory* (STAI) has recently been studied in breast cancer surgery and cesarean section, but the results are contradictory [29, 48, 56]. However, one study showed a correlation between the STAI and acute pain after cardiac surgery (*r* = 0.48, *p* < 0.01) [27].

The *Brief Measure of Emotional Preoperative Stress* (B-MEPS) is a prediction tool shown to predict acute pain after hysterectomy [57].

Fear of pain, according to the *Fear of Pain Questionnaire* (FOP-9), correlates to acute pain after thoracoscopic surgery [58, 59].

Psychological distress related to oncological surgery is a metric validated by the *National Comprehensive Cancer Network (NCCN) Distress Thermometer* and has been found to be an independent risk factor for acute pain in patients undergoing breast cancer surgery [60].

Psychological stress has been associated with acute and persistent pain after foot surgery [61] but not after orthopedic trauma [41].

One study used a machine learning algorithm on psychometric questionnaires in a large cohort of patients who underwent breast cancer surgery. This approach identified seven items in the BDI and STAI that could exclude PPOP with approximately 95% certainty [62] (Table 1).

#### 3.2.5. Evaluation of Sleep Quality

Low sleep quality has been found to predict acute pain after cesarean section as well as acute pain and longer hospital stay after breast cancer surgery [63, 64]. Additionally, low sleep quality is associated with persistent pain and opioid consumption at 6 months after hip arthroplasty [65]. In contrast, another recent study failed to repeat this association [66]. Lastly, obstructive sleep apnea syndrome (OSAS), a surrogate for poor sleep, has been found not to be associated with postoperative pain [67] (Table 1).

### 3.3. Quantitative Sensory Testing

#### 3.3.1. Evaluation of Sensitivity to Temperature-Induced Pain

Acute pain after video-assisted thoracoscopy was reported more frequently in patients with more pain on *suprathreshold cold stimulation*, whereas *the cold pain threshold* (CPT) was not predictive [37]. However, another study reported positive and negative results for CPT and suprathreshold cold stimulation, respectively, whereas heat-related QST was not associated with either APOP or PPOP [68]. On the same note, women with greater tolerance to pain induced by immersion of the hand in cold water were reported to be less likely to report persistent pain after breast cancer surgery [69]. Furthermore, no association was found between pain sensitivity to cold stimulation and pain outcomes after total knee arthroplasty [32, 70] or persistent pain after breast cancer surgery or thoracic surgery [35, 44].

Pain intensity elicited by a hot water bath has also been reported to be a predictor of APOP [50] (Table 2).

#### 3.3.2. Evaluation of Sensitivity to Pressure-Induced Pain

*The pain pressure threshold* (PPT) is the minimum applied force required to induce pain, and *pressure pain tolerance* is the maximum level of pressure tolerated. The PPT has been reported to predict acute pain after total knee arthroplasty [70], and moderate to severe APOP after pancreatic surgery, major proctological surgery, orthopedic surgery, or gynecological surgery [71, 72]. In contrast, PPT failed to predict acute pain, opioid use, or hospital length of stay after total hip or knee arthroplasty, anorectal surgery, or breast cancer surgery [73–75] and persistent pain after robot-assisted laparoscopic hysterectomy [76] (Table 2).

#### 3.3.3. Evaluation of Sensitivity to Electrically Induced Pain

*Electrical pain threshold* (EPT) was not associated with acute pain intensity after various kinds of major surgery or total knee arthroplasty [18, 70]. Similarly, no association could be established between acute pain and EPT after breast surgery [48] (Table 2). Conversely, in patients subjected to laparoscopic cholecystectomy, EPT was found to correlate with acute pain in women [77].

#### 3.3.4. Dynamic Evaluation of Pain

*Temporal summation of pain* (TSP), a surrogate endpoint for central sensitization, can predict acute pain early after total knee replacement [36]. However, in breast cancer surgery, the results are conflicting [75, 78]. *Conditioned pain modulus* (CPM) reflects endogenous descending analgesic ability. The CPM was found to predict PPOP after total knee arthroplasty [79]. However, there are several negative studies on TSP and CPM in regard to PPOP [29, 40, 48, 76, 80] (Table 2).

#### 3.3.5. Combined Techniques of Testing

In combination, high levels of preoperative pain and expected pain after surgery together with proposed extensive surgery are strong predictors for moderate to severe acute pain after day surgery [81]. Low PPT combined with high PCS can find patients who will experience APOP, with 71% sensitivity and 62% specificity [70]. In a similar manner, the combination of preoperative pain, CPM, and PCS can predict PPOP at 12 months after total knee arthroplasty [82].

The *Risk Index for Chronic Pain* (RICP) is based on assessments of preoperative pain within the proposed field of surgery, movement-evoked acute pain 5 days after surgery, other preoperative chronic pain, and female sex [83]. The RICP has been reported to predict PPOP with 75% sensitivity and 73% specificity [84]. Another study combined risk factors for PPOP, preexisting pain, depression, the expectation of pain, and age under 50 years and found that the combination of risk factors could predict PPOP [48].

Predicting persistent pain after breast cancer surgery, using a combination of risk factors was evaluated in both Danish (AUC 0.739) and Scottish (AUC 0.740) patient cohorts [85]. Machine learning has also been used to predict postoperative opioid consumption using various preoperative data. The model was shown to predict postoperative opioid requirements with approximately 70% accuracy [86] (Table 3).

### 3.4. Other Techniques of Testing

#### 3.4.1. Evaluation of Painful Routine Procedures

Using everyday procedures that cause noxious stimuli is a simple and pragmatic approach to differentiate patients with respect to pain sensitivity. Recently, pain *during subcutaneous infiltration of local anesthetic* (LA) before spinal anesthesia correlated with APOP and opioid consumption [87].

Similarly, *venous cannulation pain* (VCP) has been found to be associated with moderate to severe acute pain after various kinds of surgery, laparoscopic cholecystectomy, and laparoscopic nephrectomy; the latter two also reported on the association with postoperative opioid consumption (Table 4) [88–90].

#### 3.4.2. Evaluation With Commercial Techniques

Several new nociceptive monitoring techniques have been proposed as potential perioperative predictors of APOP (Table 4).

The *Surgical Pleth Index* (SPI) score is based on photoplethysmographic analysis of the pulse contour wave and the heartbeat interval. The maximal SPI recorded during surgical incision was found to associate with APOP and postoperative opioid consumption [91]. Conversely, more modest results have been obtained in other studies. Recently, SPI was not associated with continuous APOP scores; however, higher levels were found in patients with moderate to severe APOP. When applying an SPI cutoff of 30, a negative predictive value of 50% and a positive predictive value of 90% for APOP were found [92]. In a recent follow-up study by the same group, the overall performance of this test was poor [93]. Conversely, during liver resection, SPI correlated with acute pain in the postanesthesia care unit (PACU) (*r* = 0.63, *p* < 0.001). Furthermore, SPI was able to predict and distinguish between moderate and severe acute pain (AUC = 0.84), with sensitivity and specificity of 72% and 88%, respectively [94].

The *Pain Threshold Index* (PTI) is a neurophysiologic method used to evaluate the risk of pain in real-time. The PTI and the SPI have been reported to correlate well, and the PTI at the end of surgery can predict moderate to severe acute pain with 62% sensitivity and 91% specificity [95]. Specifically, a preoperative spectral power in the beta and gamma frequency spectrum has been reported to be associated with moderate to severe acute pain after thoracoscopic surgery [96].

Other EEG-derived indices, such as the *quantified noxious index score* (qNOX), were not found to predict acute pain in various kinds of surgical procedures [97].

The *Nociception Level Index* (NOL) is based on electrical skin conductance, heart rate variability, accelerometer, and skin temperature data provided by a finger electrode. Scores obtained immediately after skin incision at the start of surgery seem to have the highest predictive value, and scores above 20 at skin incision have been reported to predict moderate to severe APOP with 73% sensitivity and 53% specificity [98]. Notably, the NOL obtained later during surgery did not correlate with acute pain outcomes [98]. Recently, NOL levels recorded during both endotracheal intubation and surgical skin incisions were unable to predict APOP in a follow-up randomized controlled trial. However, higher time-weighted average NOL values during surgery and longer relative durations of surgery with NOL values above 25 were reported in patients with moderate to severe acute pain [99] (Table 4).

### 3.5. Genetic Testing

Data regarding genetic mapping and pain outcomes remain scarce and diverging. Single nucleotide polymorphism (SNP) in the ATP binding cassette subfamily B member 1 (ABCB1) gene is associated with acute pain after abdominal surgery [100]. Other studies have focused on genotyping and testing for alleles in catechol-O-methyl-transferase (COMT) and their association with pain after surgery [18, 101–103]. A potential association was reported between SNPs in COMT and persistent pain at six months after knee arthroplasty [103] and between SNP rs1799971 in COMT and opioid consumption after cesarean section [102].

The SNP rs11818426 in neuron navigator 3 (NAV3) has been correlated with persistent pain after hysterectomy and after total knee arthroplasty, but not in a meta-analysis of both study cohorts [104]. In another study, the SNPs rs6265 and rs1491850 in the brain-derived neurotrophic factor (BNDF) gene were both associated with persistent pain after ambulatory surgery [105].

A multivariate regression analysis of a variety of genes revealed that the inflammatory acute phase reactants (IL6, rs2069840), tumor necrosis factor (TNF, rs1800610), and C-X-C motif chemokine ligand 8 (CXCL8, rs4073) were found to be associated with 79%, 63%, and 60% lower probabilities of risk of moderate to severe acute pain, respectively, after breast surgery [106] (Table 5).

### 3.6. Biochemical Testing

*Circulating mRNA* has been reported to correlate with pain relief and to explain 30% of postoperative pain intensity in a prediction model in total knee arthroplasty [107]. Blood neutrophil/lymphocyte ratio and acute pain were associated after shoulder repair surgery [108]. Higher serum levels of *μ-opioid receptor* activity were associated with less acute pain and need for rescue analgesics after septoplasty [109]. Furthermore, measuring *angiotensin II* receptor activity in blood correlated with persistent pain after knee surgery [40]. Analysis of cerebrospinal fluid for *Substance P* and *endorphins* has also been associated with APOP [110].

*Ultraviolet B* (UVB)-induced inflammation, measured as inflammation index, was studied in patients who underwent total knee arthroplasty [111]. In this study, the authors constructed an inflammation index using the expression of six UVB-inducible genes associated with pain and concluded that preoperative inflammation correlated negatively with APOP.

## 4. Discussion

This review summarizes the current knowledge on how to predict individual risks for postoperative pain by assessing different potential factors in an effort to identify clinically valuable methods. The ability to predict postoperative pain has practical implications in making clinicians aware of, and better prepared for, pain after surgery.

### 4.1. Psychometric Testing and Evaluation of Sleep

Psychometric tools for predicting postoperative pain have been associated with both APOP and PPOP (Table 1). Although different questionnaires have been reported to enable the prediction of postoperative pain, their relevance can be questioned partly since they are all reliant on patient self-assessment [112] but also because of the heterogeneity in results as mentioned in this review. The PCS has historically shown promising results regarding its ability to predict postoperative pain [70, 113] and is still being used in research. Nevertheless, the most recent studies have obtained conflicting results on the correlation with postoperative pain and opioid use. Frankly, we see no convincing results at all, despite 18 efforts included in the review, to justify its continued use for this purpose. We believe that the unpredictability shown with the PCS demands either its refutation or usage in a different context. Recently, the ability of the HADS to predict PPOP has been investigated but not statistically confirmed, except for one study in which both the HADS anxiety and HADS depression were reported to strongly correlate with persistent pain at 3 months after hysterectomy [17]. On the other hand, the HADS score has been reported to be associated with acute pain intensity after various kinds of surgery [18–21]; thus, HADS perhaps merits more investigations regarding its use in an emergent setting. The six-item APAIS [114] was reported in the beginning of this millennium [7] to improve the prediction of APOP when combined with other tools, but more recent data are missing.

In contrast to many other psychometric tools, the Distress Thermometer is promising and not time consuming. Pak et al. evaluated psychological distress before breast cancer surgery in 956 women using the NCCN Distress Thermometer and showed that a cutoff ≥ 4 predicted APOP [60]. When used together with seven other known risk factors for APOP, the nomogram had an AUC of 0.735. Although intriguing findings have been reported in a fairly large cohort, these findings have yet to be reproduced in a prospective cohort.

Asking patients about their individual pain expectations seems promising, particularly considering the simplicity [28, 37], as do some questionnaires (PSQ and PSEQ) designed for this purpose [49, 50, 63].

Low sleep quality has been suggested to be associated with greater pain sensitivity [64]. Sleep quality based on the PSQI shows promise for preoperative screening, as the compiled study data reported in this review indicate that low-quality sleep may considerably worsen individuals' experience of postoperative pain. We anticipate a study to investigate if improved sleep quality can improve pain outcomes.

### 4.2. Quantitative Sensory Testing

Tests of experimental pain have historically been frequently linked to postoperative pain [115, 116]. Various methods for estimating pain thresholds induced by electricity, heat, cold, or pressure have all been reported to correlate with postoperative pain sensitivity. More complex dynamic methods, such as the TSP and CPM, are believed to evaluate pain processing pathways [117] and diffuse noxious inhibitory control [118]. Many recent studies on the use of QST for pain prediction have focused mainly on CPM, TSP, and pressure-induced pain (Table 2). Despite the massive amount of research performed, the results are not convincing enough to justify usage in clinical practice especially considering that its use is time-consuming and demands specific equipment and prepared surroundings.

### 4.3. Combined Techniques

Since no single preoperative test or method has been found to reliably predict postoperative pain, recent research has combined different techniques (Table 3). Several studies have come up with models that more or less associate with pain outcomes, some with fairly good results [11, 48, 119, 120]. Many, however, combine a multitude of risk factors making the clinical use complex. We are also concerned regarding publication bias since many combinations of risk factors probably were not associated with postoperative pain and thus were not elaborated on further. Despite the rationale of combining various individual risk factors to predict postoperative pain, the challenge is to achieve high enough predictive ability with as few clinically applicable factors as possible and to find the intersection between risk factors and maximum test performance. This is perhaps where machine learning and artificial intelligence come into play in the near future, since their potential reaches beyond current methodologies. However, a cautionary note must be said regarding the risk of overfitting models if too many risk factors are required to achieve high performance.

### 4.4. Other Techniques

Assessments of pain associated with standard procedures during routine preparation for surgery such as pain induced by tourniquet inflation, peripheral venous cannulation, and subcutaneous infiltration of LA have been used to evaluate pain sensitivity and predict postoperative pain intensity with promising results [88, 90, 121–123]. These techniques are simple, do not demand extra equipment, and are time-accessible, but more quality research is needed regarding its practical use.

Other new techniques, such as the SPI, the PTI, the qNOX index, the NOL index, or EEG-based techniques—all designed to quantify intraoperative nociception—have recently been proposed to predict APOP. However, it should be emphasized that results obtained with any technique based on real-time intraoperative recordings of nociception are necessarily influenced not only by individual pain sensitivity but also by the extent of surgery and potential interactions with the effects from analgesic and hypnotic drugs. The modest results obtained thus far do not currently merit investment in new intraoperative equipment for this specific purpose [91, 92, 97, 99, 124]. However, the SPI and PTI merit further investigation in clinical trials.

### 4.5. Genetics and Biomarkers

Genetic mapping is an interesting field of research since this method speaks to the very core of individualized perioperative medicine. However, current data do not suggest that this field of research is ready for clinical implementation since no genes of real interest have been identified so far. Additionally, for clinical application, sample analysis needs to be more accessible for the technique to be feasible, which is currently not achieved and argues against its use.

On the other hand, using serologic biomarkers is more accessible but brings an inherent complexity, which needs to be accounted for in order to reach its full potential in pain prediction. In the current review, none of the included studies were designed to assess the performance of a serologic biomarker as a primary outcome. At its best, association was established using group-based comparison with numeric cutoffs or correlation analyses with continuous pain outcomes. One review looked at neuroimaging-based biomarkers in pain and proposed a classification of these biomarkers based on the nature of their target variables. Here, categorization was made according to within-individual perception, between-individual sensitivity, and discriminability as well as the assessment of biomarkers for persistent pain and prospective biomarkers for persistent pain [125]. Whether this classification can be extrapolated to serologic biomarkers remains to be seen. However, it seems assessing and analyzing serologic biomarkers in a multidimensional, instead of unidimensional, manner might result in findings with higher resolution. In light of this, we anticipate future clinical trials, with a multidimensional methodology, using serological biomarkers to perhaps make it accessible for clinical practice.

### 4.6. Limitations

The risk of bias within individual original reports was not assessed, despite the original plan presented in PROSPERO. We consider this to be the biggest limitation in this systematic review. We chose to address the primary research question and include a large number of studies rather than narrowing down in preference to detailed bias analyses and presentations. To minimize the risk of selection bias, we have written, published, and followed a study protocol for literature retrieval to avoid missing relevant studies. The risk of publication bias, i.e., risk of not identifying all available data on a topic, has been addressed as seen in a total of 16 articles found and added when doing a reference screen. We still have to be open to the fact that our search strategy was potentially not as broad and inclusive as initially planned. In this review, we deliberately included a large number of studies in order to find also negative results not highlighted in a study where the main result involves a different method in order to find methods tested but with a negative result.

Although completely without the possibility of economic gain, the main author has published an original study based on a new technique reported in this review potentially leading to bias due to competing interests. However, we do not consider this to have influenced the results and conclusions of this review.

## 5. Conclusions

In conclusion, although progress is being made in the quest to find better methods for predicting pain after surgery, we still have to rely on individual risk assessments based on multiple factors added together. Most important, and readily available, are sex, age, and preoperative pain intensity. Asking patients about their believed pain sensitivity and evaluating painful events (e.g., peripheral venous cannulation) at the bedside during preparation for surgery both seem promising, as do SPI and PTI. However, other new medicotechnical devices developed for this purpose still seem to have fundamental issues to address, and psychometrics and QST have yet to show sufficient clinical value. Genetics and biomarkers are interesting but have yet to present convincing results.

For future studies, there is an urgent need for consensus regarding which parameters to use for evaluation of postoperative pain to enable comparison between studies, and ideally also consensus regarding choice of statistical methods in predictive studies.

## Figures and Tables

**Table 1:** Summary of studies covering psychometric techniques for prediction of postoperative pain and published between 2016 and 2022.

Method of testing	Kind of surgery	Number of patients	Outcomes	Statistics	Outcome measure and result (95% confidence interval)	Reference
Hospital Anxiety and Depression Scale	Knee surgery	104	APOP	Correlation	Anxiety: *r* = 0.35^†^. Depression: ns	Thomazeau 2016
	Caesarean section	1062	APOP	Logistic regression	Anxiety: OR 1.60 (1.22–2.30). Depression: ns	Borges 2016
	Mixed elective surgery	304	APOP	Correlation	Anxiety: *r* = 0.18^†††^. Depression: *r* = 0.22^†††^	Bradshaw 2016
	Knee arthroplasty	220	PPOP	Dichotomous groups^a^	Anxiety: ns. Depression: ns	Chen 2021
	Orthopedic trauma	229	PPOP	Dichotomous groups^a^	PPOP vs no PPOP, ns	Edgley 2019
	Knee/hip arthroplasty	184	APOP/PPOP	Dichotomous groups^a^	PPOP vs no PPOP, ns	Giusti 2022
	Hysterectomy	870	PPOP	Multiple logistic regression	Anxiety: OR 2.07 (1.36–2.25)	Han 2017
					Depression: OR 2.21 (1.19–4.12)	
	Gastrointestinal surgery	282	APOP	Multiple linear regression	Anxiety: *β* = 0.07 (0.01–0.14). Depression: ns	Liu 2022
	Gastrointestinal surgery	282	PPOP	Multiple linear regression	Anxiety: OR 5.28 (1.59–17.54). Depression: ns	Liu 2022
	Thoracoscopic surgery	67	APOP	Dichotomous groups^b^	Anxiety: ns. Depression: ns	Han 2021
	Knee arthroplasty	188	APOP	Linear regression	Anxiety: ns. Depression: ns	Lindberg 2017
	Breast cancer surgery	68	APOP	Multivariate logistic regression	Anxiety: OR 1.24 (1.04–1.54). Depression: ns	Nishimura 2017

Hamilton Anxiety and Depression Scale	Bariatric surgery	116	Opioid use	Correlation	Anxiety: *r* = 0.52^†††^.	Aceto 2016
					Depression: *r* = 0.52^†††^	

Pain Catastrophizing Scale	Orthopedic surgery	118	APOP	Correlation	*r* = 0.28^†^	Alokozai 2019
	Knee/hip arthroplasty	124	APOP	Correlation	THA, ns. TKA, 0.26^†^	Pinto 2018
	Knee arthroplasty	126	APOP	Correlation	*r* = 0.02, ns	Abrecht 2019
	Video-assisted thoracoscopic surgery	82	APOP	Dichotomous groups^b^	ns	Bayman 2019
	Thoracic surgery	104	PPOP	Logistic regression	OR 1.11 (0.95–1.30)	Horn–Hofmann 2018
	Laparoscopic hysterectomy	73	APOP	Correlation	*r* = 0.23, ns	Scheel 2017
	Thoracic surgery	42	APOP	Correlation	ns	Grosen 2016
	Thoracotomy/-scopy	99	PPOP	Dichotomous groups^a^	ns	Bayman 2017
	Hysterectomy	200	APOP	Binary logistic regression	1.38 (1.09 − 1.75)	Benlolo 2021
	Hysterectomy	200	PPOP	Binary logistic regression	1.04 (1.01 − 1.08)	Benlolo 2021
	Knee arthroplasty	220	PPOP	Dichotomous groups^a^	ns	Chen 2021
	Breast cancer surgery	166	PPOP	Correlation	*r* = 0.25^†††^	Dams 2022
	Knee/hip arthroplasty	184	PPOP	Multiple linear regression	*β* = 0.678^†††^	Giusti 2022
	Orthopedic trauma	303	APOP	Multivariable logistic regression	OR 0.99 (0.95, 1.03)	Edgley 2019
	Orthopedic trauma	229	PPOP	Multivariable logistic regression	OR 0.98 (0.94, 1.03)	Edgley 2019
	Breast cancer surgery	124	APOP	Multivariate logistic regression	OR 1.08 (1.02–1.14)	Habib 2019
	Knee arthrosplasty	71	PPOP	Dichotomous groups^a^	ns	Hovik 2016
	Mixed elective surgery	284	PPOP	Multiple regression	*β* = 0.153^††^	Mi 2021
	Knee arthroplasty	248	PPOP	Multivariable linear regression	Estimate 0.02 (0.002–0.05)^†^	Edwards 2022
	Thoracic surgery	107	PPOP	Dichotomous groups^a^	ns	Bayman 2017
	Cardiac surgery	100	APOP	Correlation	*r* = 0.65^††^	Tai 2021
	Mixed elective surgery	363	APOP	Multinomial logistic regression	OR 1.03 (0.98–1.09)	Vasilopoulos 2021
	Breast cancer surgery	228	APOP	ANOVA	Association^c†^	Wilson 2021
	Knee arthroplasty	60	APOP	Multivariate logistic regression	OR 1.06 (1.01–1.13)	Luna 2017

Asking the patient/expected pain	Upper extremity surgery	118	APOP	Correlation	*r* = 0.28^†^	Alokozai 2019
	Thoracosocopy	82	APOP	Correlation	*r* = 0.43^†††^	Bayman 2019
	Mixed elective surgery	304	APOP	Correlation	*r* = 0.28^†††^	Bradshaw 2016
	Knee arthroplasty	220	PPOP	Multiple logistic regression	OR 1.01 (0.99–1.02)	Chen 2021
	Breast cancer surgery	139	PPOP	Dichotomous groups^a^	Yes, if expected NRS ≤ 6^††^	Dereu 2018

Pain Sensitivity Questionnaire	Cardiac surgery	419	PPOP	Linear mixed model	*β* = 0.16 (0.06–0.26)^†††^	Bjørnnes 2018
	Breast cancer surgery	198	APOP	Multivariable logistic regression	OR 1.03 (1.01–1.05)^††^	Rehberg 2017
	Hysterectomy	200	PPOP	Correlation	*r* = 0.09, ns	Benlolo 2021
	Breast cancer surgery	139	PPOP	Dichotomous groups^a^	ns	Dereu 2018
	Knee arthroplasty	248	PPOP	Dichotomous groups^a^	ns	Edwars 2022

Pain Self-Efficacy Questionnaire	Mixed surgery	259	APOP	Logistic regressions	OR 0.96 (0.93–1.00)	Wang 2018
	Knee/hip arthroplasty	184	APOP/PPOP	Multiple regression	No predictor in model	Giusti 2022

Sleep quality	Caesarean section	245	APOP	Multivariable logistic regression	OR 2.64 (1.2–6.0)	Orbach–Zinger 2017
	Breast cancer surgery	108	APOP	Dichotomous groups^b^	Good sleep vs poor sleep^††^	Wang 2019
	Hip arthroplasty	163	PPOP	Correlation	*r* = 0.05, ns	Boye Larsen 2021
	Hip arthroplasty	52	POP	Multivariable linear regression	*β* = 0.091 (0.001–0.181), *R*^2^ = 0.35^†^	Bjurström 2021

PROMIS	Thoracotomy/-scopy	99	PPOP	Dichotomous groups^a^	3 months, ns	Bayman 2017
					6 months^††^	

WOMAC	Knee arthroplasty	220	PPOP	Multiple logistic regression	OR 0.98 (0.95–1.01)	Chen 2021

Pain Detect Questionnaire	Knee arthroplasty	220	PPOP	Multiple logistic regression	OR 1.15 (1.01–1.30)	Chen 2021

Central Sensitization Inventory	Breast cancer surgery	166	PPOP	Correlation	*r* = 0.30^†††^	Dams 2022
	Knee/hip arthroplasty	184	PPOP	Multiple regression	*β* = 0.547^††^	Giusti 2022
	Knee arthroplasty	68	PPOP	Correlation	*r* = 0.32^††^	Kim 2019

Brief Pain Inventory	Breast cancer surgery	139	PPOP	Dichotomous groups^a^	PPOP vs no PPOP^†††^	Dereu 2018
	Knee/hip arthroplasty	229	PPOP	Multivariable logistic regression	ns	Edgley 2019
	Knee/hip arthroplasty	184	PPOP	Multiple regression	*β* = 0.047, ns	Giusti 2022
	Foot surgery	49	APOP/PPOP	Dichotomous groups^a,b^	High pain trajectory vs low pain trajectory^††^	Guichard 2019
	Knee surgery	104	APOP	Correlation	*r* = 0.40^†††^	Thomazeau 2016

Beck Depression Inventory	Breast cancer surgery	139	PPOP	Dichotomous groups^a^	PPOP vs no PPOP^†††^	Dereu 2018
	Mixed elective surgery	371	PPOP	Dichotomous groups^a^	PPOP vs no PPOP^††^	Hah 2019

State-Trait Anxiety Inventory	Breast cancer surgery	139	PPOP	Dichotomous groups^a^	Trait: PPOP vs no PPOP^††^	Dereu 2018
					State: PPOP vs no PPOP^†††^	
	Cesarean section	527	PPOP	Dichotomous groups^a^	Trait: PPOP vs no PPOP, ns	Jin 2016
					State: PPOP vs no PPOP, ns	
	Cardiac surgery	100	APOP	Correlation	*r* = 0.48^††^	Tai 2021

Kessler Psychological Distress Scale	Foot surgery	49	APOP/PPOP	Dichotomous groups^a,b^	High pain trajectory vs low pain trajectory^††^	Guichard 2019
	Orthopedic trauma	303	APOP/PPOP	Multivariable logistic regression	OR 1.04 (1.00–1.08)	Edgley 2019

Center for Epidemiological Study of Depression	Cardiac surgery	98	APOP	Multivariable logistic regression	OR 1.02 (0.98–1.06)	Gohari 2022

Toronto Alexithymia Scale	Bariatric surgery	116	APOP	Correlation	*r* = 0.38^††^	Aceto 2016

Fear of Pain Questionnaire	Thoracic surgery	98	APOP	Correlation	*r* = 0.29^††^	Luo 2022

National Comprehensive Cancer Network	Breast cancer surgery	956	APOP	Multivariable logistic regression	OR 1.94 (1.16–3.30)	Pak 2022

*Note:* Significance level for regression models are shown within confidence intervals.

Abbreviations: APOP, acute postoperative pain; NRS, numeric rating scale; OR, odds ratio; PPOP, persistent postoperative pain; PROMIS, patient-reported outcomes measurement information system; WOMAC, Western Ontario–McMaster Universities Osteoarthritis Index.

^a^PPOP vs no PPOP.

^b^Moderate to severe APOP vs mild to no APOP.

^c^Higher catastrophizing in global symptoms group compared to adaptive and sensitive group.

^†^ *p* ≤ 0.05.

^††^ *p* ≤ 0.01.

^†††^ *p* ≤ 0.001.

**Table 2:** Summary of studies covering techniques for quantitative sensory testing to predict postoperative pain and published between 2016 and 2022.

Method of testing	Kind of surgery	Number of patients	Outcome	Statistics	Outcome measure and result (95% confidence interval)	Reference
Cold	VATS/thorocotomy	107	PPOP	Dichotomous groups^a^	ns	Bayman 2017
	Thoracoscopic surgery	82	APOP	Dichotomous groups^b^	NRS > 3 vs NRS ≤ 3^†^	Bayman 2019
	Breast cancer surgery	763	PPOP	Dichotomous groups^a^	Time to max NRS^†^	Lötsch 2017
					Time to NRS 10^††^	
					Sum of NRS^†^	
					Max NRS during test^††^	
	Breast cancer surgery	166	PPOP	Dichotomous groups^a^	If NRS 10 was reached^†^	Dams 2022
	Knee arthroplasty	248	PPOP	Dichotomous groups^a^	Detection cold, ns	Edwards 2022
					Pain sensitivity cold, ns	
	Thoracic surgery	111	PPOP	Dichotomous groups^a^	Cold pain tolerance, ns	Wang 2022
					Cold pain after sensation, ns	
	Knee arthroplasty	60	APOP	Dichotomous groups^b^	Cold pain threshold^†^	Luna 2017
					Cold detection threshold, ns	
					Cold pressure test, ns	

Heat	Breast cancer surgery	198	APOP	Dichotomous groups^b^	Heat pain intensity^†^	Rehberg 2017
	Breast cancer surgery	166	PPOP	Dichotomous groups^a^	Detection heat, ns	Dams 2022
					Pain sensitivity heat, ns	
	Breast surgery	139	PPOP	Dichotomous groups^a^	Pain intensity heat, ns	Dereu 2018
	Breast cancer surgery	74	APOP	Multiple linear regression	ns	Ruscheweyh 2017
	Thoracic surgery	111	PPOP	Dichotomous groups^a^	Warm detection threshold, ns	Wang 2022
					Hot pain threshold, ns	

Pressure	General surgery	1002	APOP	Multiple regression	Pressure pain tolerance^†††^	Duan 2017
					Pressure pain threshold^†††^	
	Major urological, gynecological, proctological, or orthopedic surgery	150	APOP	Correlation	Pressure pain tolerance, ns	Wolmeister 2020
					Pressure pain threshold, ns	
	Knee arthroplasty	60	APOP	Dichotomous groups^b^	Pressure pain threshold^††^	Luna 2017
	Knee arthroplasty	41	APOP	Dichotomous groups^b^	Pressure pain threshold, ns	Haghverdian 2016
	Laparoscopic hysterectomy	160	PPOP	Dichotomous groups^a^	Pressure pain threshold, ns	Lunde 2020
					Pressure pain detection, ns	
	Knee arthroplasty	220	PPOP	Dichotomous groups^a^	Pressure pain threshold, ns	Chen 2021
	Breast cancer surgery	166	PPOP	Correlation	Local mechanical detection, ns	Dams 2022
					Pain sensitivity mechanical, *r* = −0.26^††^	
					Pain sensitivity pressure, ns	
	Knee arthroplasty	50	PPOP	Dichotomous groups^a^	Pressure pain threshold, ns	Kurien 2018
	Knee arthroplasty	248	PPOP	Dichotomous groups^a^	Pressure pain threshold, ns	Edwards 2022
	Anorectal surgery	128	APOP	Correlation	Pressure pain threshold, *r* = −0.33^†††^	Luedi 2021
	Breast cancer surgery	74	APOP	Multiple linear regression	ns	Ruschewey 2017
	Knee arthroplasty	126	APOP	Multiple linear regression	Pressure pain threshold, ns	Abrecht 2019
					Pressure pain tolerance, ns	
	Breast surgery	234	APOP	Dichotomous groups^b^	Pressure pain threshold, ns	Schreiber 2019

Electrical	Knee surgery	109	APOP	Multivariate analysis	Electrical pain sensitivity, ns	Thomazeau 2016
	Knee arthroplasty	60	APOP	Dichotomous groups^b^	Electric pain threshold, ns	Luna 2017
					Electric pain tolerance, ns	
	Laparoscopic cholecystectomy	153	APOP	Correlation	Electrical pain threshold, *r* = −0.27^††^	Persson 2017
	Breast surgery	139	APOP	Dichotomous groups^b^	Electric pain threshold, ns	Dereu 2018
					Electric pain NRS 6, ns	

Temporal summation of pain	Knee arthroplasty	129	APOP	Multiple linear regression	*β* = 0.027^††^	Abrecht 2019
	Breast surgery	234	APOP	Dichotomous groups^b^	APOP vs no APOP^†^	Schreiber 2019
	Laparoscopic hysterectomy	160	PPOP	Dichotomous groups^a^	ns	Lunde 2020
	Knee arthroplasty	220	PPOP	Dichotomous groups^a^	ns	Chen 2021
	Knee arthroplasty	50	PPOP	Dichotomous groups^a^	ns	Kurien 2018
	Knee arthroplasty	248	PPOP	Dichotomous groups^a^	ns	Edwards 2022
	Breast cancer surgery	74	APOP	Dichotomous groups^b^	ns	Ruscheweyh 2017

Conditioned pain modulus	Knee arthroplasty	131	PPOP	Correlation	*r* = −0.18^†^	Larsen 2021
	Laparoscopic hysterectomy	160	PPOP	Dichotomous groups^a^	ns	Lunde 2020
	Breast cancer surgery	166	PPOP	Correlation	*r* = 0.021, ns	Dams 2022
	Breast surgery	95	PPOP	Dichotomous groups^a^	ns	Dereu 2018
	Knee arthroplasty	146	PPOP	Dichotomous groups^a^	PPOP vs no PPOP^†^	Dürsteler 2021
	Knee arthroplasty	248	PPOP	Dichotomous groups^a^	ns	Edwards 2022
	Knee arthroplasty	50	PPOP	Dichotomous groups^a^	ns	Kurien 2018
	Knee arthroplasty	126	APOP	Multiple linear regression	*r* = 0.015, ns	Abrecht 2019

Abbreviations: APOP, acute postoperative pain; NRS, numeric rating scale; PPOP, persistent postoperative pain; VATS, video-assisted thoracoscopic surgery.

^a^PPOP vs no PPOP.

^b^Moderate to severe APOP vs mild to no APOP.

^†^ *p* ≤ 0.05.

^††^ *p* ≤ 0.01.

^†††^ *p* ≤ 0.001.

**Table 3:** Summary of studies covering techniques for combined risk factor evaluation to predict postoperative pain and published between 2016 and 2022.

Method of testing	Kind of surgery	Number of patients	Outcome measure	Statistics	Outcome measure and result (95% confidence interval)	Reference
1. Preoperative pain 2. Self-anticipated APOP 3. Proposed extensive surgery	Mixed day surgery	1118	APOP	Multivariable logistic regression	Model distinction: AUC 0.82, insufficient model according to authors	Stessel 2017

1. Pressure pain threshold^††^	Total knee arthroplasty	60	APOP	Multivariate logistic regression	Model distinction: AUC 0.77 (0.65–0.89)	Luna 2017
2. Pain Catastrophizing Scale^†^						

1. Preoperative pain^††^ 2. Conditioned pain modulus 3. Pain Catastrophizing Scale^†^	Total knee arthroplasty	131	PPOP	Multiple linear regression	*R* ^2^ = 0.205	Larsen 2021

1. Preoperative pain^††^ 2. Movement-evoked APOP at 5 days^†††^ 3. Other preoperative chronic pain^†^ 4. Female gender 5. Marital status	Orthopedic, neuro-, general or abdominal surgery	167	PPOP	Multiple logistic regression	Model distinction: AUC 0.81 (0.74–0.89)	Mathes 2017

1. Preoperative pain^†††^ 2. High body mass index^†^ 3. Axillary node dissection^††^ 4. Severe APOP^†^	Breast cancer surgery	860	PPOP	Binary logistic regression	Model distinction: AUC 0.74 (0.67–0.81)-Danish cohort Model distinction: AUC 0.74 (0.65–0.83)-Scottish cohort	Meretoja 2017

1. Type of surgery 2. Medical history 3. Duration of surgery	Mixed ambulatory surgery	13,700	APOP	Multinominal logistic regression	Model predicts opioids by accuracy 70%	Nair 2020

1. Age 2. Highest APOP 3. Duration of surgery 4. Remifentanil use 5. Gender 6. Left internal mammary artery harvest	Sternotomy	174	PPOP	Multivariate logistic regression	Model distinction: AUC 0.91 (0.86–0.94)	Harrogate 2021

1. Preoperative pain^†††^ 2. History of depression^†^ 3. Self-anticipated APOP^††^ 4. Age < 50 years^††^	Breast cancer surgery	141	PPOP	Multivariable logistic regression	Model distinction: AUC 0.82 (0.73–0.91)	Dereu 2018

*Note:* Significance level for regression models and AUC are shown within confidence intervals.

Abbreviations: APOP, acute postoperative pain; OR, odds ratio; PPOP, persistent postoperative pain.

^†^ *p* ≤ 0.05.

^††^ *p* ≤ 0.01.

^†††^ *p* ≤ 0.001.

**Table 4:** Summary of studies covering other techniques to predict postoperative pain and published between 2016 and 2022.

Method of testing	Kind of surgery	Number of patients	Outcome	Statistics	Outcome measure and results (95% confidence interval)	Reference
Venous cannulation pain	Laparoscopic cholecystectomy	180	APOP	Multivariate logistic regression	OR 3.4 (1.6–7.3)	Persson 2016
	Mixed scheduled surgery	505	APOP	Multivariate logistic regression	OR 1.5 (1.0–2.3)	Persson 2019
	Laparoscopic nephrectomy	106	APOP	Multivariate logistic regression	OR 3.5 (1.3–9.3)	Peng 2020

Local anesthesia infiltration	Cesarean section	216	APOP	Correlation	*r* = 0.56^†††^	Nimmaanrat 2021

Surgical Pleth Index	Laparatomy	50	APOP	Dichotomous groups^a^	AUC 0.67 (0.51–0.83)	Jung 2020
	Mixed scheduled surgery	60	APOP	Dichotomous groups^a^	AUC 0.71^††^	Ledowski 2016
	Mixed scheduled surgery	196	APOP	Dichotomous groups^a^	AUC 0.59^†^	Ledowski 2019
	Liver resection	49	APOP	Correlation	*r* = 0.63^†††^	Park 2020

Pain Threshold Index	Laparoscopic urological surgery	76	APOP	Dichotomous groups^a^	AUC 0.77 (0.66–0.86), 62% sensitivity and 91% specificity	Wang 2020

qNOX score	Mixed scheduled surgery	144	APOP	Dichotomous groups^a^	AUC 0.50 (0.41–0.60), ns	Ledowksi 2020

Nociceptive Level Index	Mixed scheduled surgery	74	APOP	Dichotomous groups^a^	NOL post incision AUC 0.68 (0.55–0.80)	Ledowski 2021
	Gynecological laparoscopic surgery	66	APOP	Dichotomous groups^a^	AUC 0.65 (0.51–0.79)	Morisson 2022

Full-EEG	Thoracic surgery	870	APOP	Correlation	*r* = 0.84^†††^	Han 2021

*Note:* Significance level for regression models is shown within confidence intervals.

Abbreviations: APOP, acute postoperative pain; AUC, area under the curve; EEG, electroencephalography; OR, odds ratio; PPOP, persistent postoperative pain; qNOX, quantified noxious index.

^a^Moderate to severe APOP vs mild to no APOP.

^†^ *p* ≤ 0.05.

^††^ *p* ≤ 0.01.

^†††^ *p* ≤ 0.001.

**Table 5:** Summary of studies covering techniques of genetic mapping to predict postoperative pain and published between 2016 and 2022.

Method of testing	Kind of surgery	Number of patients	Outcome	Statistics	Outcome measure and result (95% confidence interval)	Reference
C3435T (*ABCB1*)	Colorectal surgery	99	APOP	Post hoc analysis bonferroni	Homozygous CC vs homozygous TT^†††^	Dzambazovska-Trajkovska 2016

rs1799971 (*OPRM1*)	Knee arthroplasty	109	PPOP	Multivariate logistic regression	OPRM1 vs no OPRM1, ns	Thomazeau 2016
rs4680 (*COMT*)					COMT vs no COMT, ns	

rs4680 (*COMT*)	Knee arthroplasty	291	PPOP	Multivariate logistic regression	COMT vs no COMT, ns	Rice 2018
rs1799971 (*OPRM1*)					OPRM1 vs no OPRM1, ns	

rs1799971 (*OPRM1*)	Cesarean section	266	PPOP	Multivariate logistic regression	COMT vs no COMT, ns	Wang 2019
rs4680 (COMT)					OPRM1 vs no OPRM1, ns	

rs11818426 (*NAV3*)	Hysterectomy orthopedic surgery	330	PPOP	Logistic regression	No genome-wide significance	RRI van Reij 2020

rs6265 (*BDNF*)	Ambulatory surgery	246	PPOP	Multivariable logistic regression	G allele vs A allele	Tian 2018
					OR 0.57 (0.39–0.85)	

rs2069840 (*IL6*)	Oncologic breast surgery	228	PPOP	Multiple logistic regression	OR 0.21 (0.07–0.63)	Stephens 2017
rs1800610 (*TNF*)					OR 0.40 (0.18–0.87)	
rs4073 (*CXCL8*)					OR 0.37 (0.15–0.89)	

*Note:* Significance level for regression models are shown within confidence intervals.

Abbreviations: ABCB1, ATP binding cassette subfamily B member; APOP, acute postoperative pain; BDNF, brain derived neurotrophic factor; COMT, catechol-O-methyl-transferase; CXCL8, C-X-C motif chemokine ligand 8; IL-6, interleukin 6; NAV3, neuron navigator 3; OPRM1, opioid receptor Mu 1; OR, odds ratio; PPOP, persistent postoperative pain; TNF, tumor necrosis factor.

^†^ *p* ≤ 0.05.

^††^ *p* ≤ 0.01.

^†††^ *p* ≤ 0.001.

## Data Availability

Data sharing is not applicable to this article as no new data were created or analyzed in this study.

## Nomenclature

APOP: Acute postoperative pain
PPOP: Persistent postoperative pain
QST: Quantitative sensory thresholds
PRISMA: Preferred Reporting Items for Systematic Reviews
HADS: The Hospital Anxiety and Depression Scale
CES-D: Center for Epidemiological Study of Depression
PCS: Pain Catastrophizing Scale
PSQ: Pain Sensitivity Questionnaire
PSEQ: Pain Self-Efficacy Questionnaire
CSI: Central Sensitization Inventory
STAI: State-Trait Anxiety Inventory
B-MEPS: Brief Measure of Emotional Preoperative Stress
FOP-9: Fear of Pain Questionnaire
NCCN: National Comprehensive Cancer Network
PSQI: Pittsburgh Sleep Quality Index
OSAS: Obstructive sleep apnea syndrome
CPT: Cold pain threshold
PPT: Pressure pain threshold
EPT: Electrical pain threshold
TSP: Temporal summation of pain
CPM: Conditioned pain modulus
RICP: Risk Index for Chronic Pain
VCP: Venous cannulation pain
SPI: Surgical Pleth Index
PACU: Postanesthesia care unit
NRS: Numeric rating scale
PTI: Pain threshold index
qNOX: Quantified noxious index score
NOL: Nociceptive Level Index
SNP: Single nucleotide polymorphism
COMT: Catechol-O-methyl-transferase
NAV3: Neuron navigator 3
